# Mitochondrial Metabolism in the Intestinal Stem Cell Niche—Sensing and Signaling in Health and Disease

**DOI:** 10.3389/fcell.2020.602814

**Published:** 2021-01-05

**Authors:** Elisabeth Urbauer, Eva Rath, Dirk Haller

**Affiliations:** ^1^Chair of Nutrition and Immunology, Technische Universität München, Freising-Weihenstephan, Germany; ^2^ZIEL Institute for Food & Health, Technische Universität München, Munich, Germany

**Keywords:** unfolded protein response, tissue homeostasis, mitochondrial quality control, Paneth cell dysfunction, metabolic integration, metabolic injury, inflammatory bowel diseases, microbial signaling

## Abstract

Mitochondrial metabolism, dynamics, and stress responses in the intestinal stem cell niche play a pivotal role in regulating intestinal epithelial cell homeostasis, including self-renewal and differentiation. In addition, mitochondria are increasingly recognized for their involvement in sensing the metabolic environment and their capability of integrating host and microbial-derived signals. Gastrointestinal diseases such as inflammatory bowel diseases and colorectal cancer are characterized by alterations of intestinal stemness, the microbial milieu, and mitochondrial metabolism. Thus, mitochondrial function emerges at the interface of determining health and disease, and failure to adapt mitochondrial function to environmental cues potentially results in aberrant tissue responses. A mechanistic understanding of the underlying role of mitochondrial fitness in intestinal pathologies is still in its infancy, and therapies targeting mitochondrial (dys)function are currently lacking. This review discusses mitochondrial signaling and metabolism in intestinal stem cells and Paneth cells as critical junction translating host- and microbe-derived signals into epithelial responses. Consequently, we propose mitochondrial fitness as a hallmark for intestinal epithelial cell plasticity, determining the regenerative capacity of the epithelium.

## Introduction

**FIGURE 1:**
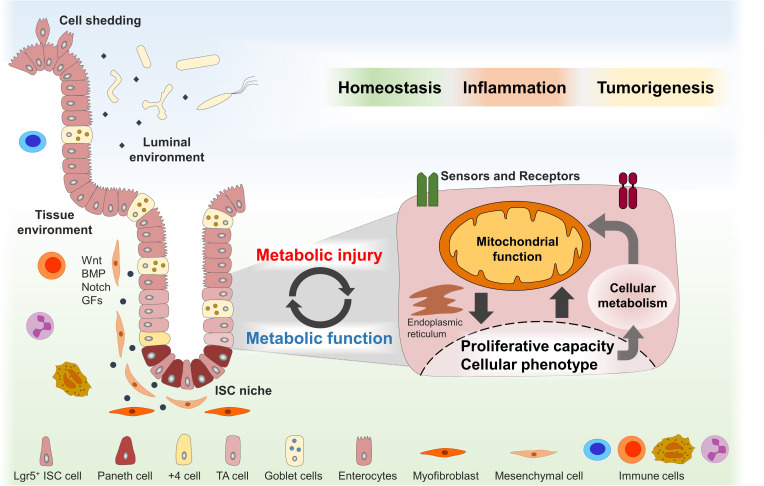
Dynamic adaptations of mitochondrial function in the ISC niche enable tissue homeostasis and might contribute to disease pathogenesis. The intestinal stem cell (ISC) niche is precisely controlled by several host-derived and luminal-derived factors and is synchronized with physiological cell shedding at the villus tip. Functional plasticity of the ISC niche is associated with dynamic adaptations of the cellular metabolism and mitochondrial function in particular and enables tissue reconstitution following inflammatory insults or wounding. Metabolic injuries, defined as disturbances of the cellular metabolism, might play a key role in the pathogenesis of inflammatory and tumorigenic disorders of the digestive tract. ISCs, intestinal stem cells; TA, transit amplifying; BMP, bone morphogenetic protein; GFs, growth factors.

Intestinal epithelial cells (IECs) not only are crucial for digestive processes but also form a physical and immune barrier for host defense (Peterson and Artis, 2014). The intestinal epithelium is an integral part of the mucosal immune system constituting a dynamic interface between the host and a complex microbial ecosystem with spatially adapted, often mutualistic mechanisms to acquire homeostasis toward the luminal milieu. The small intestine is characterized by the presence of crypt and villus structures, providing a large resorptive surface, while slightly elongated crypts build the colonic architecture. The two structurally and functionally different parts of the intestine are separated from the luminal milieu by a single layer of epithelial cells, and the complete epithelial surface of approximately 35 m^2^ is renewed every 3–5 days (Barker, 2014). Crypt-based columnar (CBC) stem cells expressing the leucine-rich repeat containing G protein-coupled receptor 5 (Lgr5) reside at the crypt base and give rise to terminally differentiated IEC subtypes (comprising enterocytes, Paneth cells, goblet cells, enteroendocrine cells, Tuft cells, and M cells) (Barker and Clevers, 2010). Interspersed between Lgr5^+^ CBCs, Paneth cells (PCs) migrate in contrast to all other cell types downward to the crypt base. Mature PCs secrete antimicrobial peptides (AMPs), such as lysozyme, defensins, angiogenin-4, and secretory phospholipase A2, to control the microbial environment. Next to protection from environmental threats, PCs further support the intestinal stem cell (ISC) niche by providing essential factors to maintain stemness, including Notch ligand (Dll4), epidermal growth factor (EGF), Wnt3, cyclic ADP ribose (cADPR), and lactate (Sato et al., 2011; Yilmaz et al., 2012; Gassler, 2017). Complementing, the underlying tissue including mesenchymal cells, fibroblasts, and nerve and immune cells supplies the ISC niche with additional signals that regulate differentiation processes (Pastula and Marcinkiewicz, 2019). Besides actively cycling ISCs, a population of slow-cycling ISCs, termed according to their position as + 4 ISCs or reserve ISCs, is located above the crypt base and is characterized by the expression of maker genes including Hopx, Lrig1, Tert, and Bmi1 (Sangiorgi and Capecchi, 2008; Montgomery et al., 2011; Takeda et al., 2011; Powell et al., 2012). These cells are resistant against acute, genotoxic stress and replace damaged CBCs in response to injury, ensuring tissue regeneration (Montgomery et al., 2011; Wang et al., 2019). ISC proliferation is synchronized with physiological cell shedding at the villus tip. Under pathological conditions, the proliferative response of ISC represents an essential mechanism for wound healing and tissue regeneration. Hence, the balance between ISC quiescence, renewal, proliferation, and differentiation is essential for maintaining homeostasis and is precisely controlled by several external and internal signals that are translated into cell-intrinsic responses. While nutrient availability as well as inflammatory cytokines and growth factors represent extrinsic influencing factors, cellular metabolism and, in particular, mitochondrial function emerge as internal targets determining the ISC niche phenotype (Rath et al., 2018; Figure 1).

Intestinal pathologies including inflammatory bowel diseases (IBD) and colorectal cancer (CRC) feature mitochondrial alterations in parallel to aberrances in SC marker expression and PC or goblet cell morphology. For example, shifts in mitochondrial metabolism are well established for cancer cells and are accompanied by ectopic expression of olfactomedin 4 (OLFM4), a marker for actively cycling ISCs that also labels a subset of CRC cells (van der Flier et al., 2009; Ashizawa et al., 2019). Consistently, changes in metabolism as well as mitochondrial genes and proteins have been described in IECs from IBD patients (Mottawea et al., 2016; Rath et al., 2018; Haberman et al., 2019) along with aberrant patterns of *LGR5* expression and reduced PC function (Wehkamp et al., 2005; VanDussen et al., 2014; Khaloian et al., 2020). Furthermore, recent data highlight the role of mitochondrial metabolism in deciding on the cellular phenotype and actively determining lineage commitment (Ludikhuize et al., 2019; Khaloian et al., 2020). In line, Paneth cell metaplasia (i.e., occurrence of PCs in the distal colon, where they are physiologically not found) seems to predispose to CRC development (Wada et al., 2005; Pai et al., 2013), and loss of mucin-producing goblet cells is an early event in intestinal inflammation (van der Post et al., 2019; López Cauce et al., 2020). The associated weakening of the colonic mucus barrier and bacterial penetration into the inner mucus layer has been proposed as a trigger for colonic inflammation as well as CRC development (Johansson et al., 2014; Coleman et al., 2018; van der Post et al., 2019). Demonstrating the relevance of mitochondrial signaling for host–pathogen interaction, *OLFM4* expression is upregulated in the gastric mucosa of *Helicobacter pylori*-infected patients (Mannick et al., 2004), while *Helicobacter pylori* vacuolating cytotoxin A causes mitochondrial network fragmentation in gastric epithelial cells (Jain et al., 2011).

Hence, we hypothesize that dynamic adaptations of mitochondrial function in the ISC niche enable tissue homeostasis in response to environmental cues and challenges and that metabolic injuries, defined as disturbances of the cellular metabolism, play a key role in the pathogenesis of inflammatory and tumorigenic disorders of the digestive tract (Figure 1). In this review, we briefly describe the role of the mitochondrial function in ISC niche homeostasis and summarize the current knowledge of external and internal signals converging on the mitochondrial function to control epithelial responses in health and disease.

## Extrinsic and Intrinsic Signals Affecting Mitochondrial Function in the ISC Niche

### Mitochondrial Metabolism and Stemness

Mitochondria are unique organelles that arose through endosymbiosis. Referred to as the “powerhouse of the cell,” mitochondria have long been reduced to their function in ATP generation. Yet, beyond energy generation through oxidative phosphorylation (OXPHOS), tricarboxylic acid cycle (TCA), and fatty acid oxidation, they contribute *i.a.* to reactive oxygen species (ROS) production, apoptosis, and immune responses and, hence, constitute a cellular signaling platform coordinating stress signaling pathways (Rath and Haller, 2012; Rath et al., 2018). Mitochondria are dynamic organelles, organized in networks physically and functionally interacting with other cellular compartments such as the endoplasmic reticulum and peroxisomes. Function and morphology of mitochondria are linked and regulated through fusion and fission, and dysfunctional mitochondria producing high levels of ROS are removed via mitochondria specific autophagy (mitophagy) (Ni et al., 2015). In the ISC niche, SCs can remain in a metabolically inactive quiescent state or an active proliferative state for self-renewal and differentiation. In general, proliferation and differentiation are thought to boost the demand for oxygen along with mitochondrial biogenesis, due to increased requirements for energy and biosynthetic processes. Cytosolic glycolysis yields pyruvate, which can be (inter)converted to lactate or be used to generate acetyl-CoA, the initial molecule fueling the TCA cycle. In turn, the TCA cycle produces substrates for OXPHOS and metabolites serving as biosynthetic precursors and signaling molecules, controlling chromatin modifications and DNA methylation, responses to hypoxia, and immune functions (Martinez-Reyes and Chandel, 2020). Interestingly, Lgr5^+^ CBCs show a higher mitochondrial OXPHOS activity compared to other differentiated epithelial cells, and a metabolic cooperation supporting stemness has been proposed between glycolytic PCs and Lgr5^+^ CBCs that use PC-derived lactate to fuel their high demand for OXPHOS (Rodriguez-Colman et al., 2017). This metabolic compartmentalization is reflected by mitochondrial morphology, with Lgr5^+^ CBCs featuring fragmented as well as fused mitochondria and PCs showing diminished mitochondrial numbers and lacking fused structures (Ludikhuize et al., 2019).

Several mouse models highlight the importance of dynamic metabolic adaptions for maintaining homeostasis in the ISC niche (Table 1). Perekatt et al. (2014) showed that IEC-specific knockout of the transcriptional repressor protein YingYang 1 (Yy1) directly regulates mitochondrial electron transport chain (ETC) genes and promotes genes involved in mitochondrial structure integrity, thus enabling OXPHOS and causing rapid SC exhaustion with Lgr5^+^ CBCs exiting the ISC niche and failing to self-renew. On the other hand, the loss of the glycolytic enzyme pyruvate kinase M2 isoform (Pkm2) in Lgr5^+^ ISCs, resulting in enhanced mitochondrial oxidative capacity and activation of mitochondrial ATP production, enhanced cancer stem cell-like functions and the development of colitis-associated colorectal cancer in mice (Kim et al., 2019). In line, the deletion of the mitochondrial pyruvate carrier (Mpc) in Lgr5^+^ ISCs, limiting pyruvate oxidation in the mitochondria, impairing TCA cycle, and promoting fatty acid oxidation, expanded the ISC compartment and proliferation in mice and Drosophila (Schell et al., 2017). Of note, mitochondrial function also seems to actively determine lineage commitment during differentiation. Lgr5^+^ ISC-specific knockout of the tumor suppressor Lkb1, a kinase regulating cell polarity, reduced oxygen consumption and altered the metabolic profile of intestinal crypts (Gao et al., 2020). This was associated with increased expression of pyruvate dehydrogenase kinase (Pdk) 4, an inhibitor of the pyruvate dehydrogenase (Pdh) complex, which controls the switch between aerobic glycolysis and OXPHOS. Inhibition of Pdh favors glycolysis and was accompanied by the induction of the transcription factor *Atoh1* (also called *Math1*), and differentiation skewed toward the secretory lineage (Gao et al., 2020). Recently, a crucial role for metabolic flexibility has also been shown for the activation of Lgr5^–^ reserve ISCs. The fructose-2,6-bisphosphatase Tigar can shift glucose metabolism toward the pentose phosphate pathway (PPP) to produce ribose-5-phosphate for nucleotide synthesis and NADPH. Thus, promoting the generation of reduced glutathione for controlling cellular ROS, Tigar was demonstrated to be indispensable for initiating reserve ISC division and crypt regeneration after lethal radiation (Cheung et al., 2013; Chen et al., 2020).

**TABLE 1:** Metabolic and mitochondrial genes impacting the ISC niche.

Gene	Function	Impact on SCs	References
*AhR* (aryl hydrocarbon receptor)	Ligand-activated transcriptional activator	Supports Lgr5^+^ ISC function	Hwang et al., 2016
*Hmgcs2* (3-hydroxy-3-methylglutaryl- coenzyme A synthetase 2)	Rate-limiting enzyme for ketogenesis	Regulates ISC regeneration and differentiation	Cheng et al., 2019
*Lkb1/Stk11* (serine/threonine protein kinase)	Controls the activity of AMP-activated protein kinase (AMPK) family members	Restricts differentiation of SCs into secretory lineage	Gao et al., 2020
*Mpc* (mitochondrial pyruvate carrier)	Imports pyruvate into mitochondria	Suppresses stem cell proliferation	Schell et al., 2017
*mTorc1* (mechanistic target of rapamycin)	Senses the nutritional status of the cell and controls cell growth and metabolism	Reduces ISC function/Lgr5 expression	Yilmaz et al., 2012; Igarashi and Guarente, 2016
*Pdk4* (pyruvate dehydrogenase kinase 4)	Regulates glucose and fatty acid metabolism	Restricts differentiation of SCs into secretory lineage	Gao et al., 2020
*Pparpgc1a* (Peroxisome proliferator- activated receptor gamma coactivator 1-alpha)	Regulates mitochondrial biogenesis, respiration, ROS accumulation, and antioxidant enzyme activities	Supports Lgr5^+^ ISC function	D’Errico et al., 2011; Bellafante et al., 2014
*Pkm2* (pyruvate kinase M2 isoform)	Glycolytic enzyme that generates ATP	Reduces self-renewal in cancer SCs	Kim et al., 2019
*Yy1* (transcriptional repressor protein YingYang 1)	Regulates mitochondrial ETC genes and promotes genes involved in maintaining mitochondrial structure integrity	Promotes stem cell renewal	Perekatt et al., 2014
*Hsp60* (heat-shock protein 60)	Mitochondrial chaperone	Supports Lgr5^+^ ISC function	Berger et al., 2016; Khaloian et al., 2020
*Parl* (mammalian mitochondrial rhomboid protease)	Controls mitophagy	Supports Lgr5^+^ ISC function	Shi and McQuibban, 2017

Cellular metabolic pathways are highly interrelated and depend on each other; thus, changes observed in distinct pathways rather represent holistic changes of the cellular metabolism (Okkelman et al., 2020). Consequently, there is also evidence that glutamine and fatty acid oxidation are altered during ISC proliferation and fate decisions (Schell et al., 2017; Okkelman et al., 2020). In particular, Hmgcs2 (3-hydroxy-3-methylglutaryl-coenzyme A [CoA] synthetase 2), the rate-limiting enzyme for ketogenesis, is implicated in regulating ISC self-renewal and secretory differentiation. Producing the ketone body β-hydroxybutyrate (βOHB), Hmgcs2 acts through the inhibition of histone deacetylases (HDAC), reinforcing Notch signaling and, in turn, promoting ISC self-renewal at the expense of PC generation. Notably, a high-fat ketogenic diet improved post-injury intestinal regeneration (Cheng et al., 2019), indicating a link between diet, the control of gene transcription via epigenetic mechanisms, and cellular functionality. Being cofactors for histone-modifying enzymes, key metabolites, such as ATP, S-Adenosylmethionin (SAM), acetyl-CoA, NAD^+^, FAD^+^, or UDP-GlcNac, similarly affect gene expression via chromatin modifications (Katada et al., 2012). This direct mechanism of converting changes in metabolism into stable patterns of gene expression supports the concept of cellular metabolism determining cell phenotypes (rather than following phenotypic changes).

In line, the mTOR pathway, a master regulator of the cellular metabolic state (Yilmaz et al., 2012; Igarashi and Guarente, 2016) as well as autophagy, not only an intracellular protein degradation pathway but also a regulator of metabolism (Cadwell et al., 2008; Riffelmacher et al., 2018), plays critical roles in the ISC niche.

### Mitochondria as Targets and Regulators of Wnt Signaling

The tight interrelation between mitochondrial function and intestinal stemness is further highlighted by the fact that Wnt signaling, one of the key pathways regulating the ISC niche (Spit et al., 2018), impacts cellular metabolism and *vice versa*; mitochondrial signaling affects the Wnt pathway (Delgado-Deida et al., 2020). These mechanisms have been investigated particularly in the context of CRC. Hyperactivation of the Wnt pathway is believed to be an initiating and driving event in CRC pathogenesis (Schatoff et al., 2017), and at the same time, cancer cells feature a metabolic shift from OXPHOS to aerobic glycolysis, known as the “Warburg effect.” In line, activation of the canonical Wnt pathway and its target gene cMyc impacts glucose and glutamine metabolism and regulates genes involved in the biogenesis of ribosomes and mitochondria (Dang et al., 2009). The Wnt-triggered induction of aerobic glycolysis is mediated by upregulation of a broad array of glycolytic enzymes, including lactate dehydrogenase A (Ldha), Pdk 1 and 4, Pkm2, hexokinase 2 (Hk2), phosphofructokinase (Pfkm), and monocarboxylate transporter 1 (Mct1), but also the glucose transporter Glut1 (Dang et al., 2009; Pate et al., 2014; Cha et al., 2020). On the other hand, Wnt signaling directly suppresses mitochondrial respiration by inhibiting the expression of cytochrome C oxidase (*Cox*) subunits (Lee et al., 2012), complementing the Warburg-like reprogramming of cellular metabolism that has been suggested as a pro-proliferative oncogenic signal (Lecarpentier et al., 2019; La Vecchia and Sebastian, 2020).

Creating a bidirectional cross-talk loop between mitochondrial metabolism and Wnt signaling, mitochondria-derived signals have been demonstrated to affect the Wnt pathway by several mechanisms. For instance, Costa et al. (2019) identified a mitochondria-Wnt signaling axis in which mitochondrial ATP is required to maintain endoplasmic reticulum homeostasis, in turn sustaining Wnt signaling. Along this line, intestinal-specific depletion of the mitochondrial transcription factor A (*Tfam*) decreased mitochondrial respiration, subsequently reducing Wnt signaling and tumor formation in Apc-mutant mice (Wen et al., 2019). Additionally, pharmacological depletion of the metabolite SAM, a methyl donor controlling one-carbon metabolism, has been shown to inhibit the canonical Wnt pathway (Albrecht et al., 2019), which might be partly mediated via redirecting mitochondrial one-carbon fluxes (Cuyas et al., 2018). A different mechanism has been characterized by Bernkopf et al. Upon mitochondrial stress, the mitochondrial phosphatase Pgam5 is cleaved by the rhomboid protease presenilin-associated rhomboid-like protein (Parl), inducing Pgam5 translocation to the cytosol, and resulting in dephosphorylation and thus stabilization of ß-catenin, a key mediator of Wnt signaling. This cell-intrinsic activation of the Wnt pathway, in turn, replenished the mitochondrial pool and restored mitochondrial homeostasis (Bernkopf et al., 2018). Collectively, these data implicate that regulation of intestinal stemness and metabolism converge on the level of Wnt signaling and underline the importance of mitochondria-derived signals for intestinal homeostasis.

### Mitochondrial Homeostasis, Stress Signaling, and Stemness

**FIGURE 2:**
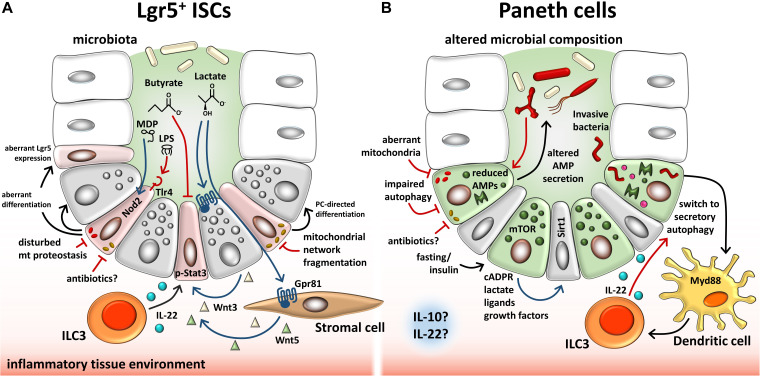
Overview of endogenous and exogenous factors involved in regulating the ISC niche. **(A)** Described effects on Lgr5^+^ ISCs. Microbiota-dependent lactate binds to the cell surface receptor Gpr81 on PCs and stromal cells, which, in turn, secrete Wnt factors that support ISCs. ILC3s secrete IL-22, modulating proliferation of ISCs and progenitor cells via phosphorylation of Stat3. Contrarily, microbiota-derived butyrate inhibits ISC proliferation. Bacterial LPS and MDP bind to TLR4 and NOD2, respectively, on ISCs, resulting in enhanced apoptosis (TLR4) or cytoprotection of ISCs. Invasive bacteria and their toxins cause mitochondrial network fragmentation, a process associated with induction of PC-directed differentiation. In line, disturbances of mitochondrial proteostasis cause mitochondrial dysfunction associated with dysfunctional PCs and reduced Lgr5 expression. **(B)** Described effects on PCs. Dysbiosis in IBD is associated with reduced and/or altered AMP secretion by PCs, resembling a dysfunctional phenotype. Impaired autophagy/mitophagy and degenerating mitochondria are associated with this PC phenotype. Infection of PCs causes a switch to secretory autophagy (causing diffuse lysozyme expression) via Myd88-dependent bacterial sensing in dendritic cells resulting in the activation of ILC3 and IL-22 secretion. Fasting and insulin signaling impact PCs via mTOR-mediated mechanisms including cADPR production. Paracrine-released cADPR activates Sirt1 and promotes ISC function. Similarly, PC-derived metabolites, ligands, and growth factors support ISCs. Furthermore, antibiotics and an inflammatory tissue environment, in general, have the potential to alter PC behavior and stemness through mitochondrial-mediated signals in the intestine. PCs, Paneth cells; ISCs, intestinal stem cells; LPS, lipopolysaccharide; MDP, muramyl dipeptide; ILCs, innate lymphoid cells; Tlr, toll-like receptor; Nod2, nucleotide-binding oligomerization domain-containing protein 2; Stat3, signal transducer and activator of transcription 3; AMP, antimicrobial peptide; mTOR, mammalian target of rapamycin; Myd88, myeloid differentiation primary response 88; cADPR, cyclic ADP ribose; Sirt1, sirtuin1.

In parallel to metabolism, mitochondrial quality control (MQC) systems, including signaling to maintain mitochondrial proteostasis (mitochondrial unfolded protein response) and control of mitochondrial dynamics, i.e., mitochondrial biogenesis, fusion, fission, and mitophagy, are essential to intestinal stemness (Berger et al., 2016; Boyle et al., 2018; Deng et al., 2018; Ludikhuize et al., 2019; Figure 2A). MQC ensures mitochondrial functionality and, hence, enables metabolic adaptations. An example for the interaction of MQC and metabolism is the mammalian mitochondrial rhomboid protease Parl, a regulator of mitophagy that is activated by Pdk2, a key regulator of metabolic plasticity upon depletion of mitochondrial ATP (Shi and McQuibban, 2017). Evidence for a role of MQC in ISCs comes from *Drosophila*, where ISC proliferation is controlled by the *Drosophila* PGC-1 homolog, a master regulator of mitochondrial biogenesis (Rera et al., 2011), and ISC differentiation is dependent on mitochondrial fusion (Deng et al., 2018). Consistently, Foxo transcription factors together with Notch signaling seem to converge on the regulation of mitochondrial networks; knockdown of *Foxo1/3* in IECs resulted in increased mitochondrial fragmentation along with reduced mitochondrial respiration and steered ISC differentiation to secretory IEC subtypes, goblet cells, and PCs (Ludikhuize et al., 2019). Similarly, but in the context of mitochondrial proteostasis, an IEC-specific loss of prohibitin 1 (Phb1), exerting chaperon functions in the inner mitochondrial membrane, regulating *i.a*. fusion events and supporting ETC functions, was associated with mitochondrial dysfunction, activation of mitochondrial undfoled protein response (MT-UPR), PC defects, and increased levels of *Math1* (Jackson et al., 2020). In line, IEC-specific deletion of the MT-UPR-associated transcription factor activating transcription factor (Atf) 4 caused PC dysfunction and spontaneous enterocolitis in mice, along with disturbances in amino acid metabolism due to reduced glutamine uptake via Slc1a5 (Hu et al., 2019). Further dissecting the role of MT-UPR in intestinal stemness, we demonstrated that IEC-specific loss of heat shock protein (Hsp) 60, the major chaperone of the mitochondrial matrix, induced MT-UPR signaling, diminished mitochondrial respiration, and concomitantly abrogated intestinal stemness and proliferation (Berger et al., 2016). Using a model in which Hsp60 deletion was selectively induced in Lgr5^+^ ISCs, we could furthermore illustrate that Hsp60-deficiency drives Lgr5^+^ ISC differentiation into a dysfunctional PC phenotype (Khaloian et al., 2020). Of note, in *C. elegans*, Wnt signaling has been shown to propagate mitochondrial stress across tissues, serving as mitokine to trigger MT-UPR in a cell-non-autonomous fashion (Zhang et al., 2018), indicating a direct link between the major pathway determining intestinal stemness (Wnt) and MT-UPR.

### Mitochondria and Paneth Cell Function Under Pathological Conditions

Remarkably, mitochondrial morphology and (dys)function closely correlate with PC phenotypes observed under disease conditions. Disturbances of AMP packaging into granules and alterations in the granule exocytosis pathway are reflected by disorganized and reduced numbers of cytoplasmic granules as well as a diffuse cytoplasmic lysozyme expression, and represent a common phenomenon under inflammatory conditions like IBD and infections, but also upon injury, defects in autophagy, or MT-UPR induction (Figure 2B). Furthermore, the PC phenotype can correlate with the differentiation status, in line with mitochondrial function. Mechanistically, the exocytosis pathway, PC maturation, and Wnt signaling are functionally linked via Rab8a, a small GTPase facilitating exocytotic cargo movements essential to these processes (Das et al., 2015). A key feature of the ISC niche is its enormous plasticity and redundancy of cells that can replenish the pool of actively cycling ISCs, and PCs contribute to epithelial regeneration under stress conditions. Upon radiation-induced tissue injury involving Lgr5^+^ CBC loss, or chemically induced inflammation, PCs can leave their terminally differentiated state to acquire stem-like properties and switch to a proliferative phenotype to ensure regenerative processes (Roth et al., 2012; Schmitt et al., 2018; Yu et al., 2018). Contrarily to dedifferentiation, a lack of differentiation can also occur under pathologic situations. Immature PCs, so called intermediate cells, appear during inflammation and infection and are characterized by coexpression of goblet cell and PC markers (Vidrich et al., 2005; Walsh et al., 2009). Both dedifferentiation as well as incomplete differentiation yield a PC phenotype comprising the above-mentioned characteristics of aberrant AMP secretion.

### Paneth Cell Phenotype in IBD

There is extensive evidence for altered cellular metabolism, mitochondrial dysfunction, perturbed mitochondrial dynamics, mitochondriopathy, and activation of mitochondrial stress signaling including MT-UPR in IBD, and genetic risk loci have been mapped to mitochondrial function-associated genes (Rath and Haller, 2012; Rath et al., 2018; Denson, 2020; Jackson and Theiss, 2020; Mancini et al., 2020). IBD includes two distinct idiopathic pathologies, Crohn’s disease (CD) und ulcerative colitis (UC). While the mucosal inflammation in UC is restricted to the colon, CD can involve different areas of the gastrointestinal tract but predominantly affects the terminal ileum and is characterized by a transmural inflammation (Podolsky, 1991). The current paradigm for the pathogenesis of IBD is a dysregulated interaction between the intestinal microbiota and the mucosal immune system in genetically predisposed individuals, whereby onset, progression, and recurrence of disease are most likely triggered by unknown environmental agents (Mayer, 2010). This sets the intestinal epithelium, goblet cells, and particularly PCs in the focus of disease development, conferring a function as a barrier and a mediator between microbiota and host-derived signals. PCs have even been suggested as sites of origin for CD (Adolph et al., 2013), and PC-specific dysfunction can initiate intestinal inflammation. For example, mice with a conditional deletion of caspase-8 in the intestinal epithelium (*Casp8*^Δ*IEC*^) lack PCs and show reduced numbers of goblet cells, along with spontaneous development of inflammatory lesions in the terminal ileum and a high susceptibility to colitis (Gunther et al., 2011). Targeting specifically PCs by using a *defensin 6 alpha* promotor-driven Cre expression, both loss of the ER stress-associated protein Xbp1 (Adolph et al., 2013) and the mitochondrial chaperone Phb1 (Jackson et al., 2020) resulted in secretory granule alterations in PCs and spontaneous ileitis. Yet, lineage-specific ablation of PCs does not result in intestinal inflammation (Durand et al., 2012), and PC dysfunction might be secondary to intestinal inflammation in TNF^ΔARE^ mice (Schaubeck et al., 2016). Due to dysregulated expression of tumor necrosis factor (Tnf), TNF^ΔARE^ mice develop a CD-like ileitis that is dependent on microbial composition and characterized by diminished expression of PC-derived AMPs (Schaubeck et al., 2016) and impaired mitochondrial function in the ISC niche (Khaloian et al., 2020). However, aberrances in PC granules are already present in mildly inflamed tissues from TNF^ΔARE^ mice (Khaloian et al., 2020). Together with data from mice with an IEC-specific deletion of an X-linked inhibitor of apoptosis protein (*Xiap*), which only develops ileitis upon certain bacterial triggers despite the presence of PC alterations (Gopalakrishnan et al., 2019), these results underline the notion of a complex CD pathogenesis involving environmental cues as well as erroneous signals arising from dysfunctional PCs.

PC defects are frequent in CD and have been extensively investigated (Wehkamp and Stange, 2020). PCs directly sense the microbial environment and release their AMP-filled granules to shape the microbiota and prevent microbial invasion; thus, the observed dysfunctional PC morphology has been linked to IBD-associated dysbiotic changes of the microbiota, comprising decreased species richness and altered bacterial composition (Vaishnava et al., 2008; Eom et al., 2018). Accordingly, patients with ileal CD show diminished PC alpha-defensin-production (Wehkamp et al., 2005), a class of AMPs that has been proposed to control numbers of segmented-filamentous bacteria (SFBs) (Salzman et al., 2010), epithelial-attaching bacteria that have been detected in IBD patients (Finotti et al., 2017). Studies in mice on human CD-relevant genetic risk variants, such as *XBP1* (endoplasmatic reticulum stress response), *IGRM* and *ATG16L1* (autophagy), or *NOD2* (bacterial sensing), highlight the role of PCs in CD pathology. Mutations in these genes impair AMP production and secretion in PCs, and the cumulative number of CD-associated *NOD2* and *ATG16L1* risk alleles is associated with the proportion of dysfunctional PCs in CD patients (Cadwell et al., 2008; Kaser et al., 2008; Adolph et al., 2013; VanDussen et al., 2014). Importantly, in mice with hypomorphic expression of *ATG16L1*, impaired granule exocytosis is paralleled by degenerating mitochondria and changes in the transcriptional profile of metabolic genes in PCs (Cadwell et al., 2008). Accordingly, Irgm1-deficient mice display marked alterations of PC granule morphology, along with swollen mitochondria and impaired mitophagy (Liu et al., 2013). Confirming the importance of autophagy and associated mitophagy for epithelial integrity, IEC deficiency in the essential autophagy protein Atg5 results in a similar PC phenotype (Cadwell et al., 2008), and a cytoprotective function of Atg16l1 during TNF-mediated necroptosis was linked to the role of autophagy in promoting mitochondrial homeostasis (Matsuzawa-Ishimoto et al., 2017). Furthermore, the mitophagy protein NIX was found to be upregulated in IBD patients and experimental colitis, probably clearing damaged and dysfunctional mitochondria (Vincent et al., 2020). *Vice versa*, specifically inducing mitochondrial dysfunction and MT-UPR in IECs via Hsp60-deletion or in PCs via Phb1-deletion resulted in the appearance of dysfunctional PCs and a concomitant reduction of OXPHOS capacity (Berger et al., 2016; Jackson et al., 2020). In line, treatment of intestinal organoids with the OXPHOS inhibitor oligomycin yielded the same, dysfunctional PC phenotype and an associated loss of Lgr5 expression (Khaloian et al., 2020).

### Intestinal Stem Cell Niche in IBD: A Paradigm for Metabolic Injury

So far, the role of PCs in maintaining ISC homeostasis has been underappreciated in the context of CD-associated PC dysfunction. However, diminished PC defensin production is associated with reduced Wnt signaling (Wehkamp et al., 2007; Beisner et al., 2014), and vesicular trafficking influences Wnt signaling capacities in both ligand-producing and ligand-receiving cells (Feng and Gao, 2015). During aging, PCs contribute to the decline in ISC function and reduced regenerative capacity by production of Notum, an extracellular Wnt inhibitor, thus not only promoting but also actively down-regulating intestinal stemness (Pentinmikko et al., 2019). Moreover, IBD-relevant ER stress signaling interferes with Wnt signaling and leads to a loss of Lgr5^+^ ISCs (Heijmans et al., 2013; van Lidth de Jeude et al., 2017), and ER dysfunction might impair Wnt factor maturation in general, as newly synthesized Wnt proteins need to be lipid-modified in the ER by the acyltransferase Porcupine (Takada et al., 2006). In IBD, the recurrent inflammatory episodes evoke repeated wounding/healing processes, and tissue responses to maintain/restore the intestinal barrier require mitochondrial function-dependent cellular phenotypic changes that are likely reflected by alterations of the ISC niche. Consequently, in a mouse model of CD-like ileitis (TNF^ΔARE^ mice), inflammation severity could be correlated to the number of dysfunctional PCs as well as to reduced stemness, findings that were paralleled by MT-UPR induction and mitochondrial dysfunction in ileal crypts under inflammatory conditions (Khaloian et al., 2020). Most importantly, characterizing tissue sections from CD patients, we could retrieve the results from the animal model, and furthermore, ISC niche alterations in CD patients in remission were found to be predictive for early endoscopic recurrence (Khaloian et al., 2020). Ileal crypts derived from TNF^ΔARE^ mice failed to develop into organoid structures; yet, the addition of dichloroacetate (DCA), an FDA-approved drug shifting the cellular metabolism from anaerobic glycolysis to mitochondrial respiration, was able to rescue the phenotype. Of note, subsequent withdrawal of DCA did not affect organoid growth, providing a proof-of-concept that metabolic reprogramming might be a therapeutic target in IBD (Khaloian et al., 2020). These data suggest that mitochondrial dysfunction and the associated aberrant phenotype of PCs and ISCs are an early event in CD pathology; hence, further research is needed to clarify if these alterations are initiating events or already compensatory responses to maintain tissue homeostasis. We propose that intrinsic defects in cellular metabolism (= metabolic injury) cause epithelial dysfunction evoking attempts of the ISC niche to reconstitute normal tissue architecture and function. *Vice versa*, functional adaptations of the ISC niche are initiated by extrinsic signals and are associated with mitochondrial alterations. Failure to resolve metabolic injuries, however, might contribute to inflammatory processes and neoplasia (Figure 1).

### Mitochondrial Function as Target of Cytokines

Inflammatory processes and tissue restitution in the intestine depend on immune cells and their secreted factors (Xue and Falcon, 2019). Immune cells affect Wnt signaling in the intestine, directly targeting the ISC niche to drive tissue responses. Especially macrophages have been identified as important sources of Wnt factors (Cosin-Roger et al., 2019), but also as targets of Wnt signals affecting their polarization (Yang et al., 2018; Malsin et al., 2019). Additionally, IBD-relevant cytokines such as interferon gamma, IL-6, and TNF have been shown to regulate antimicrobial peptide secretion from PCs, ISC activation, and proliferation (Bradford et al., 2017; Jeffery et al., 2017), as well as mitochondrial metabolism (Hahn et al., 2014). IL-22 and IL-10 are key cytokines in maintaining immune homeostasis and promoting tissue healing. Both cytokines play profound roles in IBD pathology (Ouyang and O’Garra, 2019) and provide evidence for immune cell-derived signals to modulate mitochondrial function for controlling epithelial responses. IL-22 protects ISCs against genotoxic stress, infection, chemotherapeutics, and immune-mediated damage (Hanash et al., 2012; Aparicio-Domingo et al., 2015; Gronke et al., 2019). Acting via activation of a signal transducer and an activator of transcription 3 on both Lgr5^+^ stem cells (Lindemans et al., 2015) and transit-amplifying cells (Zha et al., 2019) and downstream modulating pathways such as Wnt, Notch, and ER stress response (Zha et al., 2019; Powell et al., 2020), IL-22 consequently impacts on intestinal organoid growth (Zwarycz et al., 2019). Of note, both IL-22 and the immunomodulatory cytokine IL-10 have been shown to act on mitochondrial homeostasis and metabolism. Treatment of intestinal organoids with IL-22 altered the gene expression of glucose metabolism-associated genes Hk2 and Pck1 and lipid metabolic process (Chen et al., 2019), whereas in adipocytes, IL-22 modulated lipogenesis, lipolysis, and β-oxidation, and in rat insulin secreting cells, IL-22 conferred protective functions on mitochondrial membrane potential (Hu et al., 2017). For IL-10, a more complex regulation of metabolism has been described, and this metabolic reprogramming was suggested to mediate IL-10’s anti-inflammatory effect on macrophages. In this setting, IL-10 was shown to inhibit lipopolysaccharide-induced glucose uptake and glycolysis, promote OXPHOS, suppress mammalian target of rapamycin (mTOR) activity via induction of the mTOR inhibitor DDIT4, and enhance the elimination of dysfunctional mitochondria by mitophagy (Ip et al., 2017). It is attractive to hypothesize that IL-22 and IL-10 exert a protective function on the intestinal epithelium via modulation of the cellular metabolism, controlling IEC proliferation and tissue regeneration, and that this metabolism-targeted signaling could be a target for future therapeutic interventions.

### Mitochondria as Metabolic Integrators of Microbial Signals

Functional plasticity (e.g., regenerative response) and barrier integrity of the intestinal interface depend not only on the coordinated contribution of host-related factors but also on the microbial milieu (Jackson and Theiss, 2020). Certain pathogens are reported to interact with mitochondria to impact IEC homeostasis. For instance, infection with *Listeria monocytogenes* caused mitochondrial network fragmentation in a human IEC line, consistent with *Helicobacter pylori* vacuolating cytotoxin A inducing mitochondrial fragmentation in gastric epithelial cells (Jain et al., 2011). Of note, *Helicobacter pylori*-infected patients display enhanced expression of the stem cell-marker *OLFM4* (Mannick et al., 2004), substantiating the interrelation of microbial signaling, mitochondrial activity, and ISC niche. Interestingly, infection with invasive bacteria causes PCs to switch to secretory autophagy, an autophagy-based alternative secretory pathway that is characterized by diffuse lysozyme expression (Figure 2), hence resembling the dysfunctional PC phenotype observed during injury and inflammation (Bel et al., 2017). Even to a larger magnitude, the normal intestinal microbiota modulates ISC niche function and host metabolism through direct contact or release of products/metabolites (Everard et al., 2014; Ocansey et al., 2019), and studies in germ-free mice demonstrated a profound effect of the microbiota on IEC maturation and differentiation (Wichmann et al., 2013; Macpherson and McCoy, 2015), with PC and goblet cell numbers significantly increased in microbiota-harboring mice (Johansson et al., 2015; Schoenborn et al., 2019). Of note, the microbiota composition and their functions differ along the gastrointestinal tract (Crespo-Piazuelo et al., 2018), and IECs are extensively adapted to their specific microbial milieu. For example, colonocytes use microbiota-derived short-chain fatty acids (SCFAs) as their major energy source, while small intestinal enterocytes predominantly utilize glucose and glutamine for energy generation (Rath et al., 2018). Underlining the link between metabolism and epithelial responses, SCFAs promote growth of intestinal organoids and regulate genes involved in energy metabolism and PGC1α, a master regulator of mitochondrial biogenesis (Lukovac et al., 2014; Tan et al., 2014; Park et al., 2016). With regard to IBD, numerous reports have indicated shifts in microbial composition associated *i.a.* with reductions in SCFA-producing bacteria (Caruso et al., 2020). Specifically, colonocytes from germ-free mice display a diminished activity of PDH, shifting metabolism from OXPHOS to glycolysis and concomitantly showing impaired cell cycle progression, effects that can be rescued by supplementation of butyrate (Donohoe et al., 2012). Next to host-derived metabolic effectors, the two best described microbiota-derived metabolites impacting on intestinal stemness are lactate and butyrate, and both have been shown to act as an energy substrate as well as a signaling molecule via G-protein coupled receptors (Gprs) to modulate epithelial homeostasis (Donohoe et al., 2012; Rodriguez-Colman et al., 2017; Lee et al., 2018; Liu H. et al., 2018; Table 2). Butyrate can additionally inhibit histone deacetylase activity, in turn conveying a growth-inhibiting effect on colonic ISC via Foxo3. Yet, differentiated colonocytes metabolize butyrate, preventing ISCs from butyrate exposure, and thus, the colonic crypt architecture has been suggested to form a metabolic barrier (Kaiko et al., 2016). In contrast, microbiota-derived lactate, next to PC-derived lactate serving to support OXPHOS in ISCs (as mentioned earlier) (Rodriguez-Colman et al., 2017), has been shown to activate Gpr81 either on PCs or stromal cells, both resulting in Wnt factor production and expansion of Lgr5^+^ ISCs (Lee et al., 2018; Figure 2). Additionally, the bacterial metabolite hydrogen sulfide (H_2_S) has been shown to target mitochondrial functions in the intestinal epithelium. In IECs, H_2_S is capable of inducing genotoxic damage, and elevated levels of H_2_S can impair OXPHOS by inhibiting complex IV of the electron transfer chain (Ijssennagger et al., 2016; Saint-Georges-Chaumet and Edeas, 2016). Indicating disease relevance, an integrated microbiota and metabolite profile analysis has recently linked Crohn’s disease activity to bacterial sulfur metabolism (Metwaly et al., 2020). Moreover, in IBD, an adverse microbiota–host interaction has been reported for H_2_S, with increased abundance of sulfate-reducing bacteria (i.e., H_2_S-producers) on the microbial side and a decreased expression of mitochondrial proteins involved in hydrogen sulfide detoxification on the host side (Mottawea et al., 2016).

**TABLE 2:** Endogenous and exogenous metabolic effectors targeting the ISC niche.

Substance	Function	Impact on SCs	References
Lactate	Energy substrate for ISCs G protein coupled receptor ligand	Supports Lgr5^+^ ISC function Enhances Wnt3 production by PCs and stromal cells produced by PCs and the microbiota	Rodriguez-Colman et al., 2017; Lee et al., 2018
Butyrate	Energy substrate for colonocytes G protein coupled receptor ligand Histone deacetylase inhibitor	Reduces ISC function	Kaiko et al., 2016; Donohoe et al., 2011
Galactose	Activates OXPHOS through the Leloir pathway	Substitution of glucose with galactose enhanced crypt formation	Rodriguez-Colman et al., 2017
cADPR	Paracrine effector activating calcium signaling	Promotes ISC function produced by PCs	Igarashi and Guarente, 2016
Insulin	Induces mTOR in PCs	Shifts the balance of Lgr5^+^ SC differentiation versus self-renewal, favoring differentiation	Yilmaz et al., 2012
Rapamycin	Inhibition of mTOR	Increases frequency of ISC and PCs	Yilmaz et al., 2012
DCA	Inhibition of glycolysis	Enhances crypt formation	Khaloian et al., 2020; Rodriguez-Colman et al., 2017
Oligomycin	Inhibition of OXPHOS	Reduces ISC function	Khaloian et al., 2020
Rotenone	Inhibition of OXPHOS	Reduces ISC function	Rodriguez-Colman et al., 2017

Direct interactions between bacterial products and Lgr5^+^ ISCs have been described for the pattern recognition receptors toll-like receptor (Tlr)4 and Nod2, activated by the bacterial ligands lipopolysaccharide and muramyl dipeptide, respectively (Figure 2). While Tlr4-signaling reduced proliferation and induced apoptosis in ISCs, contributing to the pathogenesis of necrotizing enterocolitis (Neal et al., 2012; Sodhi et al., 2012; Naito et al., 2017), the activation of Nod2 during irradiation-induced stress resulted in ISC protection against ROS cytotoxicity via stimulation of mitophagy (Levy et al., 2020). The distinct roles of bacterial sensing are most likely context and localization (apical versus basolateral) dependent. It might be interesting to investigate if similarly, activation of Tlr9, which recognizes mitochondrial DNA released under stress conditions (Hu et al., 2017), alters ISC niche behavior.

In addition to innate immune mechanisms integrating microbial signals, IECs are equipped with various sensors, receptors, and transceptors like purinergic receptors (ATP receptors), chemosensitive receptors (AhR), and olfactory (OR) receptors (Zietek and Rath, 2016; Inami et al., 2018; Marinelli et al., 2019; Kotlo et al., 2020) to surveil the cellular environment and adapt the metabolic state accordingly. For instance, ORs, G protein-coupled receptors serving as chemosensors, respond to microbial metabolites including SCFAs, in turn impacting on whole-body metabolism by activating the release of incretin hormones from enteroendocrine cells (Kim et al., 2017) and also modulate tissue responses to colitogenic stimuli (Kotlo et al., 2020). In line, aryl hydrocarbon receptor (Ahr) signaling is a well-described pathway modulated by endogenous and microbiota-derived compounds such as butyrate and derivatives of tryptophan including indole and kynurenines (Brinkmann et al., 2019; Marinelli et al., 2019). The Ahr is a ligand-activated transcription factor that can be found in the cytosol as well as inner mitochondrial membrane (Casado, 2016), and an interaction of Ahr and mitochondrial function has been suggested (Brinkmann et al., 2019). In particular, kynurenines are capable of altering cellular respiration and metabolic pathways (Hwang et al., 2016), and by maintaining ISC homeostasis and modulating Notch signaling, Ahr has been shown to enhance epithelial barrier function and protected from inflammatory damage (Liu Z. et al., 2018; Metidji et al., 2018). Interestingly, PCs expressing indolamin-2,3-dioxygenase (Ido) 1, converting the amino acid tryptophan into kynurenines, have been identified to enhance tumor formation by promoting an immune-tolerant microenvironment via local tryptophan depletion. Ido^+^ PCs are also present in normal murine crypts, and it was suggested that microbiota-derived signals via IFN gamma and Stat1 enhance the numbers of Ido^+^ PCs as local immunosuppressors to prevent aberrant immune cell activation in response to bacteria (Pflugler et al., 2020). Hence, metabolites derived from intestinal microbes and IECs under homeostasis and stress conditions might act to shape intestinal immune responses. These data offer additional (metabolic) roads to go for improving IBD treatment via reconstitution of the broken immune tolerance toward the indigenous microbiota through metabolic modifications.

### Antibiotics Targeting Mitochondria and Stemness

There are numerous approaches for targeting the microbiota and reversing dysbiosis in various diseases, including fecal microbiota transplantation, the use of pre- and probiotics, nutritional interventions, and application of antibiotics (Caruso et al., 2020). Yet, antibiotics might be a so far unrecognized modifier of the ISC niche via their effect on mitochondria. Repeated exposure to antibiotics, in particular during childhood, is associated with an increased risk for IBD (Shaw et al., 2010, 2011). The common notion is that disturbances of the microbiota might have long-term negative effects on microbiota composition or that early inflammatory episodes might be a sign for later disease susceptibility (Ozkul et al., 2020). Recently, it was shown that Abx treatment in combination with a high-fat diet impairs epithelial mitochondrial function, alters intestinal microbial composition, and exacerbates intestinal inflammation (Lee et al., 2020). However, Abx can also directly target the host. In the context of sepsis, it was shown that doxorubicin conferred protection independently of the pathogen burden by activating the DNA-damage response and autophagy in the lung (Figueiredo et al., 2013). In particular, owing to their bacterial heritage, mitochondria are effectively targeted by several classes of antibiotics (Abx). Clinically relevant doses of bactericidal Abx were shown to cause mitochondrial dysfunction characterized by reduced mitochondrial membrane potential and ATP production, lowered respiration, and increased fission in human epithelial cell lines. This was accompanied by enhanced ROS production, resulting in oxidative tissue damage (Kalghatgi et al., 2013; Kohanski et al., 2016) and apoptosis induction (Arimura et al., 2012). In line, Abx treatment of colon and breast cancer cells resulted in mitochondrial dysfunction and mitophagy induction (Esner et al., 2017; Boyle et al., 2018). Mechanistically, this was dependent on the AMP-activated kinase (AMPK) and mTOR signaling pathway. Additionally, tetracyclins were reported to evoke mitonuclear protein imbalance by inhibiting mitochondrial translation, induce MT-UPR, and reduce OXPHOS capacity in both *Drosophila* and mice (Moullan et al., 2015). Hence, Abx might not only cause a shift in the microbiota toward a disadvantageous composition with, e.g., reduced numbers of lactate acid-producing (and therefore stemness-promoting) bacteria but also directly affect the ISC niche via mitochondrial impairment, causing disturbances in tissue homeostasis.

## Conclusion and Outlook

In recent years, mitochondria emerged as new frontiers in intestinal tissue homeostasis and disease pathogenesis. Mitochondrial fitness in the ISC niche plays an essential role in maintaining IEC homeostasis and determines PC phenotype and stemness, and extrinsic as well as intrinsic factors converge at this junction. We hypothesize that failure of mitochondrial functionality in the epithelium leads to chronic activation of MT-UPR and epithelial dysfunction, and that this metabolic injury causes aberrant tissue responses reminiscent of intestinal reconstitution responses. To date, it remains largely elusive which factors targeting mitochondrial function control epithelial cell regeneration in response to barrier or metabolic disruption, and how these signals contribute to either healing and tissue homeostasis, or under which circumstances dysregulation of these mechanisms favors chronic inflammation or tumorigenesis.

Of note, enhancing intestinal stemness through genetic modifications has been shown to protect against T-cell-mediated (Bayrer et al., 2018) and chemically induced colitis (Koren et al., 2018), providing a proof-of-concept for targeting the ISC niche to prevent inflammatory flares. The control of the proliferative capacity is furthermore of particular interest in the context of inflammatory processes, as constantly enhanced proliferation rates observed in inflammation-related wounding in IBD seem to predispose to neoplastic alterations (Rhodes and Campbell, 2002). Interestingly, 5-amino salicylic acid, often the first treatment option for newly diagnosed IBD patients, has been shown to revert adverse changes in mitochondrial biogenesis and metabolism-associated genes as well as mitochondrial metabolism evoked by exposure of mice to pre-IBD risk factors (Lee et al., 2020). First attempts of direct mitochondria-targeted therapies using DCA as a metabolic modulator or P110, a small peptide inhibitor of mitochondrial fission, yielded promising results in restoring the ileal ISC niche (Khaloian et al., 2020) and reducing chemically induced colitis in mice (Mancini et al., 2020), respectively. Thus, a better molecular understanding of signals and mediators in regenerative tissue responses and resolution of metabolic injuries is critical to develop clinically relevant therapeutic interventions focusing on the ISC niche, a novel strategy for combating intestinal diseases.

## Author Contributions

All authors listed have made a substantial, direct and intellectual contribution to the work, and approved it for publication.

## Conflict of Interest

The authors declare that the research was conducted in the absence of any commercial or financial relationships that could be construed as a potential conflict of interest.
